# Editorial: Gene Regulation From the X-Chromosome During Development and Disease

**DOI:** 10.3389/fcell.2020.00272

**Published:** 2020-04-23

**Authors:** Montserrat C. Anguera, Bernhard Payer, Céline Morey

**Affiliations:** ^1^Department of Biomedical Sciences, School of Veterinary Medicine, University of Pennsylvania, Philadelphia, PA, United States; ^2^Centre for Genomic Regulation (CRG), The Barcelona Institute of Science and Technology, Barcelona, Spain; ^3^Universitat Pompeu Fabra (UPF), Barcelona, Spain; ^4^Université de Paris, Epigenetics and Cell Fate, CNRS, Paris, France

**Keywords:** Xist RNA, dosage compensation, X-reactivation, XCI escape, X-chromosome inactivation (XCI), sex differences

X-chromosome Inactivation (XCI), which is the dosage compensation mechanism utilized by female mammals, is a hallmark example of epigenetic gene regulation. The memory of a transcriptionally silent X chromosome, chosen at random between maternal and paternal Xs during embryogenesis, is maintained with each round of cell division. Thus, XCI is responsible for the cellular mosaicism of female mammals (Figure 1). This Research Topic on XCI and gene expression from the X chromosome deals with various aspects, at the molecular level, of regulatory processes that form the inactive X, which has strong implications for understanding sex differences during development and disease.

**Figure 1:**
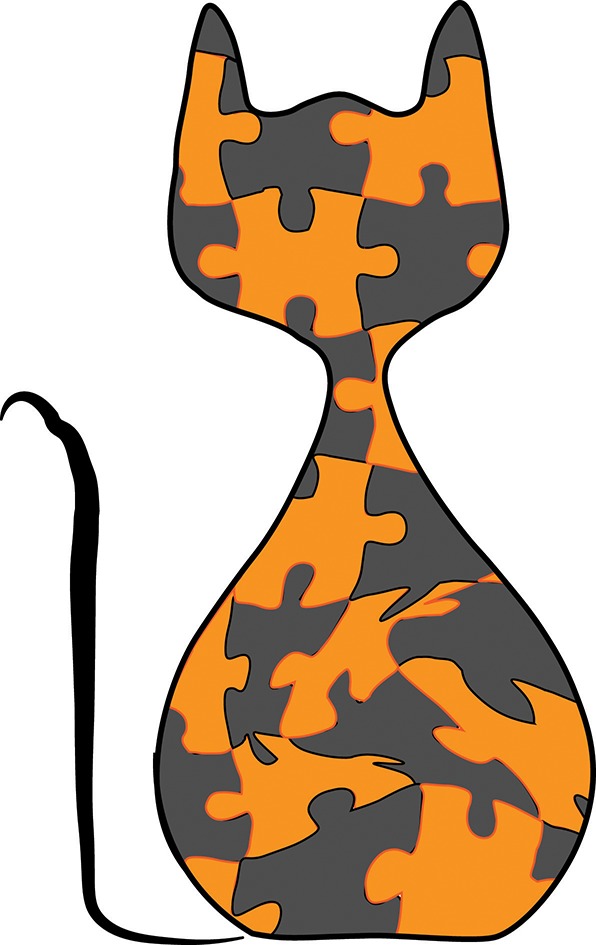
The mixed coat color of tortoiseshell cats has been the classic example for the mosaicism caused by random X-chromosome inactivation in female mammals. This cartoon symbolizes the fascinating scientific puzzle of X-inactivation, which scientists have been trying to solve for many years, since its first description by Lyon (1961).

One important aspect of the developmental regulation of XCI is its tight coupling with the exit from pluripotency. In their review, Wang and Bach revisit the role played by RLIM/RNF12 in the molecular coupling of exit from pluripotency and XCI initiation. RLIM is a E3 ubiquitin ligase which targets the pluripotency factor REX1 for proteasome degradation, thereby preventing REX1-mediated repression of *Xist* upon cell differentiation and initiation of random XCI. RLIM is also a maternal factor involved in the first wave of imprinted XCI that characterizes mouse pre-implantation stages. The authors discuss possible modes of action of RLIM in these totipotent contexts suggesting, notably, that it may facilitate imprinted XCI and control the subcellular localization of REX1 during pre-implantation development.

XCI is controlled in *cis* by the X-inactivation center (*XIC*), a locus on the X chromosome harboring a cluster of long non-coding RNA genes including *Xist* and some of its regulators *Tsix, Ftx*, and *Jpx*, and the protein-coding gene *RLIM/RNF12*. The clustering of these genes within topologically associated domains (TADs) functions to coordinate expression and regulation of *Xist* during XCI. Barbara Migeon presents a new hypothesis about gene organization of XCI factors in a Mini Review. She notes that *trans*-acting XCI regulators cluster together on autosomes, similar to the *cis*-acting factors, which cluster in the *XIC* region on the X. This suggests that the clustering of autosomal XCI regulators might facilitate their coordinated regulation. These observations could guide the path for future experiments like testing the interactions of these genomic regions, perturbations of regulatory elements, or relocation of these genes to ectopic locations.

The inactive X is structurally distinct from the active X (and the autosomes) because it consists of two large “mega-domains” separated by the microsatellite *DXZ4* boundary region. Bansal et al. review recent findings on the changes to spatial organization during the formation of the inactive X chromosome during early female embryonic development. While *DXZ4* and other X-linked tandem repeats (*FIRRE* and *ICCE*) are well-conserved and form long-range looping interactions, deletions of these repeats have mild effects on XCI maintenance and escape. The authors propose that evolutionary conservation of X-linked tandem repeats may have occurred for *cis* and *trans*-acting regulation on the active X.

While the vast majority of X-linked genes are subject to XCI, some genes resist the global silencing of the X chromosome. The molecular mechanisms responsible for XCI escape are an enduring enigma in the field. Allelic analyses of RNA-seq data, both at the single-cell level and on cell populations showing biased X-inactivation, have revealed extensive variability with escape genes across cell types and tissues. Fang et al. review the molecular mechanisms involved in XCI escape, addressing, notably, the contribution of nuclear organization, lncRNAs, chromatin features, and insulation mechanisms promoted by CTCF/YY1. Posynick and Brown take an original angle of examining XCI escape in the light of evolution. Both the number and chromosome distribution of XCI escapees varies between mammalian species, such as the PAR (pseudoautosomal region) of the human X-chromosome, which is enriched with escapees. The realization that some escapees are organized in clusters that tend to coincide with TADs may shed some light on the mechanisms underlying insulation and help to explain the differences in escapee landscapes between species. XCI escape may also contribute to sex differences for disease development, such as autoimmunity.

Recent advances in pluripotent stem cell reprogramming and single-cell methods have enabled addressing the reverse process of XCI—how the inactive X chromosome can be awakened by X-chromosome reactivation. In their review article, Talon et al. give an overview of the occurrences of X-reactivation *in vivo* during mouse and human development, *in vitro* during induced pluripotent stem cell (iPSC)-reprogramming, and following fusion between somatic cells and pluripotent stem cells. It is still unclear how epigenetic and transcriptional regulators remodel the structure and epigenetic state of the inactive X for reactivation. It also remains to be explained why some genes along the X are reactivated faster than others, which is important for revealing the underlying molecular mechanisms of the reactivation process itself.

It is well-established that adult tissues have sex-specific gene expression differences whose origins could be genetic (arising from the sex chromosomes) and/or influenced by hormones and also the environment. The minireview by Deegan and Engel examines the developmental origins of sex differences of gene expression. The authors propose that epigenetic modifications originating from the sex chromosomes act genome-wide after fertilization to establish sex-specific epigenetic modifications that persist into adulthood. While these modifications first appear during early development, the impact on transcription can occur later during development and often persist into adulthood.

In sum, the manuscripts that comprise this Research Topic provide a broad overview of the latest research investigating XCI and the developmental origins of sex-differences involving X-linked genes.

## Author Contributions

All authors contributed equally in editing this Research Topic and in writing of this editorial, and approved it for publication.

## Conflict of Interest

The authors declare that the research was conducted in the absence of any commercial or financial relationships that could be construed as a potential conflict of interest. The handling editor declare a shared affiliation with one of the authors CM.
